# RF Line-Element Filters for Structural-Health-Monitoring Applications

**DOI:** 10.3390/s22228908

**Published:** 2022-11-18

**Authors:** Idris Musa, John Hedley

**Affiliations:** School of Engineering, Newcastle University, Newcastle-upon-Tyne NE1 7RU, UK

**Keywords:** structural health monitoring (SHM), line-element filter, mutual coupling, passive sensors, displacement sensing, crack sensing

## Abstract

RF-based sensors are an attractive option for structural-health-monitoring applications, due to the ease of access of interrogating such sensors. However, in most work, only scalar quantities are measured, giving no indication of the direction of strain or displacements. In this paper, a novel approach to displacement sensing is presented, in which relative displacements are tracked in all three degrees of freedom. The sensor design is based on a pair of coupled line-element filters whose frequency-dependent forward-power transfer is sensitive to relative positions between the two filters. Multiple features in the S_21_ parameter are used to differentiate displacement direction. Gold-based devices were fabricated on quartz substrates, and characterised through vector-network-analyzer measurements. Results demonstrate uncoupled sensitivities of −1.41 MHz/mm, −1.74 MHz/mm and 12.23 MHz/mm for x, y and z displacements, respectively.

## 1. Introduction

The inspection and monitoring of structures, known as structural health monitoring (SHM), is generally dependent on a network of carefully distributed sensors or observations that measure the structure over time without impairing the system functionality [1]. When the sensing devices are part of the structures, wireless sensors are preferred to reduce cost for both installation and maintenance of the monitoring systems. Recently, SHM is leaning towards intelligent monitoring systems to investigate both the characteristics and the evolution of damaged structures and to see how these structures perform under loading effect, stress, and degradation, together with material aging. SHM systems find significant application in areas such as railway, naval, aerospace, and other relevant industries to inspect structures during forced-vibration testing or natural excitation from the environment. To acquire the complete assessment of the condition of the structure, a high number of suitably located observations or sensor measurements are taken over the structure.

Several conventional means for observation and measurement are generally used for SHM. These may be non-contact, for example, imaging-based techniques to visually inspect the structure, or sensors in physical contact with the structure, such as accelerometers and piezoelectric transducers. Contact sensors require attachment to the surface undergoing observation, and they are then interrogated. The unity of the sensor with the underlying material means that the detected signal is modified in a way that can be correlated to the condition or motion of the structure. Common contact-sensor choices include strain gauges and fibre-optic sensors, because of their comparative cheapness and efficiency. Fibre-optic sensors rely on cable connections for their acquisition; however, these systems are found to have a high cost of installation and an unsuitable functionality when many devices are required over a large area [2]. Strain-gauge measurements are another popular choice for SHM. These are particularly appealing when arranged in a wireless configuration, thereby overcoming access difficulties at the required point of measurement. The capability of performing long-distance measurements and handling fast time-varying phenomena such as vibration monitoring, makes such strain-gauge sensors appealing for many practical applications [3]. Comprehensive reviews on a range of SHM sensing-technologies is given in [4,5]. A key specification in developing detailed SHM, in which a high density of sensors is needed, is the ability to power and interrogate such sensor networks easily. This makes radio-frequency (RF) sensing-techniques an attractive option, as a hardwired connection is not required for powering the sensors or retrieving the required data. Consequently, a number of researchers are investigating this RF-technology approach for the inspection and deformation monitoring of structures.

The motivation for this work is to develop a novel high-frequency line-element filter arrangement, designed to act as a displacement-vector sensor for SHM applications where both magnitude and direction of displacement is determined. The principle of the approach is to design a filter with multiple features in the transmission passband, and then to couple two filters; monitoring of these features then leads to a displacement measurement in three translational degrees of freedom. In this work, characterisation is carried out through a wired connection; however, the device design is suited to implementation in wireless sensor-networks.

The paper is arranged as follows. Section 2 details a range of RF-sensor approaches reported in the literature and targeted at SHM applications. Section 3 and Section 4 describe the sensor design and experimental arrangement, to characterise the sensor performance. Section 5 presents the results, and Section 6 discusses these results with respect to similar work in the field, as reported in Section 2. The paper concludes with a summary in Section 7.

## 2. Literature Review

Originally designed as identification tags, radio-frequency identification (RFID) has evolved to become wireless identification and sensing platforms (WISP), incorporating computing and sensing capabilities [6]. DiGiampaolo et al. [3] employed a strain-gauge approach, coupled with a battery-powered RFID chip for sensor integration and wireless transmission. The approach allows for dynamic measurements and temperature compensation, and is suited to large-scale monitoring through a star-topology network. The authors identify the fact that that a main area for improvement is extending battery life, potentially through energy harvesting. Zhang et al. [7] proposed that RFID breakage triggered strain-sensor-based systems coupled with building information modelling (BIM), to enable structural-condition monitoring of civil infrastructures. Pre-set strain thresholds were used to identify weak points in the structure, and alert engineers. The system identified locations where the pre-set strain threshold had been surpassed, allowing alert signals to be sent to the engineers. The RFID-sensor deformation-monitoring was validated via hybrid testing of a truss structure under monotonic loading. Sunny et al. [8] also utilised low-frequency RFID tags, in this case to monitor corrosion. Their work looked to optimise the wireless power-transfer to the RFID, to maintain a high-quality factor for the sensor. Both maximum signal-value and rate of change of signal were used to determine permeability and conductivity changes of the metal substrate. Zhang et al. [9] utilised commercial 13.56 MHz RFID tags to measure the corrosion of steel over a twelve-month period. By extracting the real and imaginary parts of the complex impedance, and performing principal component analysis on this data, the work demonstrated the suitability of this approach as a position-independent corrosion-monitoring technique.

Surface-acoustic-wave (SAW) devices have shown promise in both sensitivity and the ability to interrogate wirelessly. Saitoh et al. [10] designed a passive shear-horizontal-mode SAW RFID device. By using these edge-deflected delay lines, the device was shown to be suitable for a range of remote-sensing applications, including liquid and strain sensing. Humphries et al. [11] designed a passive SAW sensor on a YZ-cut lithium niobate substrate with an orthogonal-frequency-coding frequency bank used for identification of the device. Via a cantilever experimental-setup, device performance was assessed with a VNA. The device was firstly interrogated using a direct-wired connection, and then wirelessly to demonstrate remote access. Results were comparable. The temperature sensitivity of the lithium niobate was significant and noted by the authors as an area that needs addressing.

Sensors based on antenna designs naturally lend themselves to wireless operation, as these devices have no specific sensing unit because the antenna is itself the sensor. One option to interrogate these sensors is by tracking the resonant frequency of such devices. Cho et al. [12] developed a novel passive antenna strain-sensor in which a receiving patch-antenna is connect to a transmitting patch-antenna via a matching network. The transmitting antenna’s resonant frequency is twice that of the receiving antenna. The antenna was bonded to a structural surface, and strains in the structure produced a shift in the resonant frequency. The sensor was also able to identify crack formation under the sensor up to crack displacements of 5.5 mm, at which point the sensor breaks. Yi et al. [13] developed a passive wireless-antenna sensor for applications in strain measurement and crack sensing. Deformations induced in the antenna result in a shift in the resonant frequency of the antenna. A wirelessly powered RFID chip was incorporated into the design to allow for elimination of background noise from the signal. Strain measurements ranging from 20 µε to 10,000 µε were demonstrated, together with a tracking of the propagation of a crack. Yi et al. further extended this work by considering what dielectric changes need to be considered when modelling such devices [14]. Kuhn et al. [1] focused on reducing overall sensor-size for RFID-based strain-sensors by utilizing a planar inverted-F antenna (PIFA)-geometry. Through parametric design and experimental verification, the work optimized the antenna geometry and showed the importance of matching the resonant frequency to the operating frequency of the chip to obtain good linearity and a high signal-to-noise ratio for such sensors. Khalifeh et al. [15] presented a pair of wireless radio-frequency corrosion-sensitive resonators. The sensors were arranged such that the first was sensitive to metal loss through degradation whilst the second was sensitive to the corrosion potential with respect to a reference electrode. The system is being developed for the monitoring of metal corrosion and degradation of organic coatings in coastal regions and underwater environments. Kim et al. [16] looked at printing stretchable RFID tags using silver-based nano inks aimed at biomedical applications. The pressure applied during stamping and the subsequent heat annealing strengthen the sensors, resulting in the possibility of strain measurements of up to 7% on PDMS substrates.

Another option for wireless interrogation is to monitor either the power threshold to activate the device or the measurement of the reflected power from the device. Caizzone et al. [17] monitored crack propagation with the use of two passive RFID antennas placed at either side of a crack. The backscattered power from the T-matched tags was used to measure the crack displacement. Although demonstrating good sensitivity, the range for high-sensitivity measurements was limited to around ±1 mm, although ways to improve on this are suggested. Cazeca et al. [18] illustrated the viability of using standard RFID tags to develop budget wireless displacement-sensors, suitable for infrastructure monitoring. Their sensor was constructed by splitting an RFID tag into two parts, comprising a tag antenna and the chip-loop, which can be displaced with respect to each other. The displacement was then shown to be a function of transmitted power with a high degree of linearity. A wireless embroidered integrated-RFID-enabled strain sensor was demonstrated by Hasani et al. [19], in which the sensor was interrogated using two different power levels. Due to the nonlinear impedance of the RFID chip, the difference between the responses indicated the sensing signal with background noise eliminated. The technique was demonstrated on a stretchable fabric at strains of up to 10%, and characterised using a bistatic-radar approach. Similarly, Merilampi et al. [20] demonstrated high strain-measurements on stretchable polyvinyl chloride and fabric substrates using RFID tags with screen-printed antennas, using silver ink. Strain sensing through amplitude of the backscattered signal was employed. High strain-measurements on skin were presented by Rakibet et al. [21]. In this work, an epidermal passive RFID strain sensor on a flexible barium-titanate-loaded polydimethylsiloxane (PDMS) substrate was used. Transmission-threshold power was used to interrogate the sensor, and strains of up to 10% were measured. The increase in permittivity associated with barium titanate loading in the substrate allowed the effect of tag detuning to be emphasized as a function of stretch, thereby increasing sensitivity.

Several researchers have investigated the use of antenna sensors for determining direction of strain. Tata et al. [22] utilized a rectangular microstrip patch-antenna with changes in antenna resonant-frequency giving a measure of applied strain. The system was experimentally verified using a loaded cantilever beam. Two resonances were used, and the sensitivity of both frequencies to x and y strains determined. This work originally incorporated cabling to access the sensor, but further development [23] led to a wireless solution for interrogation. Daliri et al. [24] investigated wireless sensing using a circular microstrip patch-antenna (CMPA). The advantage of a CMPA is the ability to measure strain in any direction, although it does not differentiate between the directions the strain is being applied to. The method was experimentally confirmed on both conductive and dielectric substrates. It was shown that alignment of the interrogating horn antenna significantly affected sensor sensitivity. In order to determine strain direction, Li et al. [2] arranged multiple patch-antenna sensors in a rosetta arrangement. The work showed that high sensitivities were obtained for longitudinal strain-measurements; however, for slotted patch-antennas there was a significant cross-sensitivity to transverse-strain effects as compared to folded patch-antenna designs.

A table summarising the performance of these sensors is given in Section 6. A comprehensive review of passive antenna-based sensor systems for applications in SHM is given by Zhang et al. [25].

## 3. Design and Fabrication

**Figure 1:**
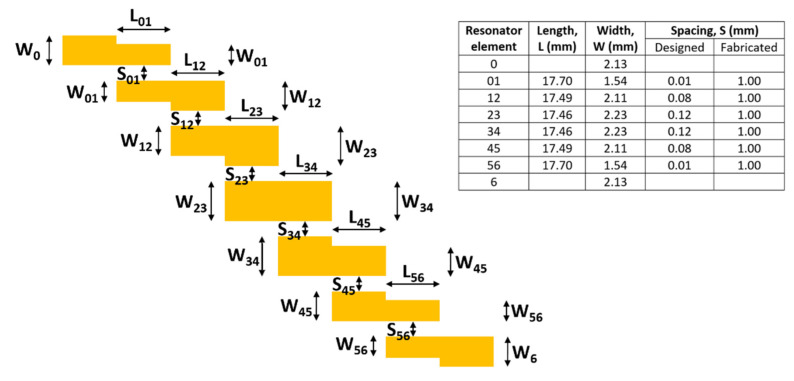
Schematic for the design of the 5th-order microstrip band-pass filter. Geometric parameters for a filter with a 2.5 GHz central frequency are given in the adjacent table.

The sensing element employed for this sensor is an RF microstrip band-pass filter. This parallel-coupled microstrip filter uses half-wavelength lines to represent each open-ended microstrip resonator. They are positioned in such a way that the adjacent resonators are parallel to each other along half of their length. This parallel arrangement provides a large coupling between each resonator. The general structure of the parallel-coupled microstrip band-pass filter is shown in Figure 1. These coupled microstrip-lines are characterised by their characteristic impedances, together with the effective dielectric constants of their odd and even modes.

The microstrip band-pass-filter design-parameters were an impedance of 50 Ω, a central frequency of 2.5 GHz and a fractional bandwidth of 20%, with 1 dB Chebyshev filter ripple-loss. The substrate choice is a 1 mm thick quartz substrate of relative permittivity 3.8. This planar filter was designed following a similar procedure described by Dipak et al. [26] in which corresponding J-inverter values were calculated, which were used to determine the even- and odd-mode characteristic impedances and geometric ratios for the elements, see [27] for more details. From these normalized prototype-element-values, the final filter geometry was determined, as shown in Figure 1. The filters were to be fabricated on standard-sized quartz microscope slides of dimensions 75 mm by 25 mm. However, the design shown in Figure 1 has an overall length of 122 mm. The design was modified by flipping the geometry lengthwise at the midpoint of the filter, forming a more compact V-shaped design. In addition, due to manufacturing tolerances, the minimum element-spacing was fixed at 1 mm. The performance of this final filter-design was verified by simulation on the finite element package ANSYS, using the high-frequency structure simulator.

**Figure 2:**
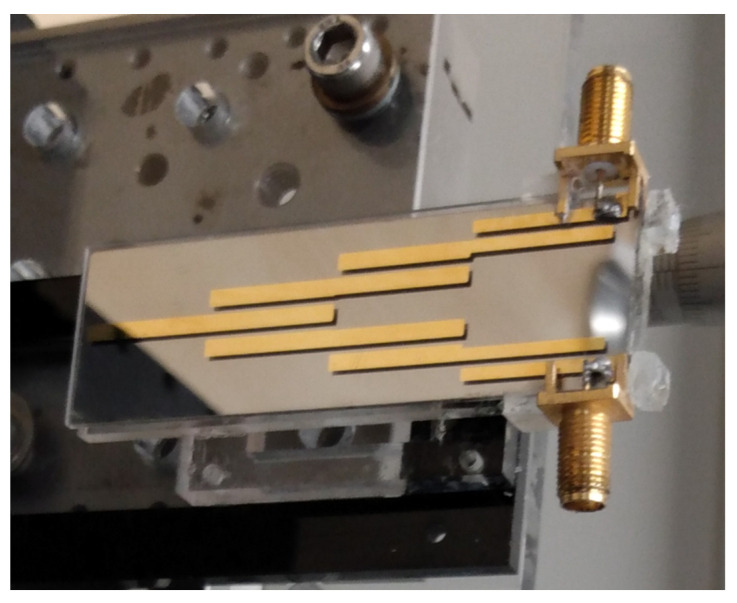
A fabricated gold microstrip band-pass filter on a quartz substrate.

Filters were fabricated at INEX Microtechnology Ltd. The mask design was produced on Autodesk Inventor software and then exported to a CNC micro milling machine. A 150 mm diameter aluminium disk was CNC machined to form a shadow mask for the fabrication process; gaps between filter elements were limited to a minimum of 1 mm width, to maintain the structural integrity of the mask during milling. The filter design was cut through the disk, with a 0.5 mm recess also being cut into the disk in order to align the quartz slide with these features. Photoresist was used as an adhesive to secure the quartz slides during the evaporation process. A 50 nm titanium adhesion-layer was first evaporated through the mask and onto the slide, followed by 300 nm of gold. The disk was then flipped and a 50 nm titanium/300 nm gold layer evaporated onto the back surface of the slides, to act as a ground plane. A total of 6 slides were attached to the disk during evaporation. On completion of the evaporation, the slides were detached from the shadow mask, using acetone. A pair of SMA connectors were soldered onto each filter and the filter was then fixed to an acrylic plate, for mounting. An image of one completed filter is shown in Figure 2.

## 4. Experimental Setup

**Figure 3:**
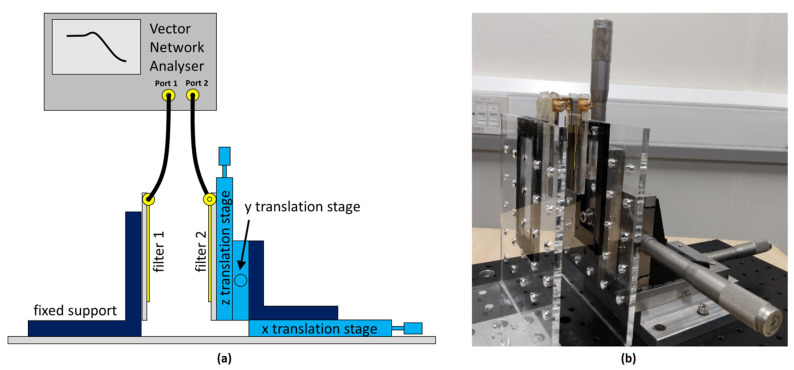
The band-pass filters and measurement set-up used for displacement-sensing characterisation. (**a**) Schematic of setup, (**b**) actual hardware implementation (VNA not connected).

To determine the suitability of the devices for SHM, the following arrangement was set up. Filter one was attached to a stationary holder, whilst filter two was attached to a 3-axis translation stage. The two filters were then placed facing each other in close proximity. The y- and z-axis origins were aligned for both filters, with the x-axis being initially offset by 8 mm, to make room for the SMA connectors. This was classed as the zero position, and all displacements given are with respect to this starting point. A schematic of the setup is shown in Figure 3, together with the actual experimental arrangement. A Rohde Schwarz ZVL3 vector network analyser (VNA) was calibrated using a ZV-Z135 calibration kit and then connected to the filters, port 1 being connected to the input side of filter 1 and port 2 to the output side of filter 2. The scan range for the VNA was set from 0 Hz to 3 GHz, with an impedance of 50 Ω, the power set at 1 dBm, the number of points to 4000 and with an averaging factor of 49.

To simulate the motion or deformation of a structure of interest, the manual translation stages were each sequentially adjusted from 0 mm to 15 mm in 1 mm steps, before being returned to their starting position. At each position, S_11_ scans were taken over the scan range. The procedure was repeated, to obtain S_21_ traces. As shown in Figure 3, S_11_ was taken across filter 1, with filter 2 acting simply as a shielding plane. For S_21_ measurements, the measurements were taken from the input side of filter 1 to the output side of filter 2. In the arrangement used, x displacements moved the filters further apart and z displacements offset the filters across their relatively long lengths, whereas y displacements offset filters across their widths and corresponding narrow track-spacings. It was therefore anticipated that y axis translations would produce the most noticeable variations in the traces obtained.

**Figure 4:**
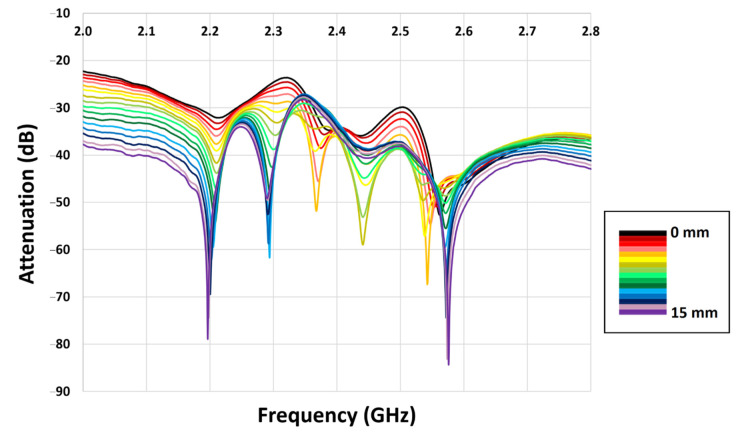
Variation in S_21_ as filter 2 is displaced from filter 1 in the y direction.

Results for S_11_ showed little variation, as a displacement was introduced between the two filters, whereas S_21_ showed good sensitivity to displacement in all three axes. Figure 4 shows the obtained S_21_ traces for displacements of filter 2 in the y direction. Similar traces were obtained for displacements in the x and z directions.

From the traces, six regions of interest were identified in which prominent notches were evident, as described in Table 1. To determine the position of each of these features, a Matlab script was used. The data for each of the frequency ranges of interest were loaded into Matlab and a program automatically determined the deepest attenuation in that data set. The program then fit a 5th order polynomial to the surrounding 21 data points, to determine position and attenuation of each mode. All fits were visually inspected during this process. Where fits were deemed inaccurate due to the depth of the feature, a spline fit was used to estimate peak position and attenuation.

## 5. Results

**Figure 5:**
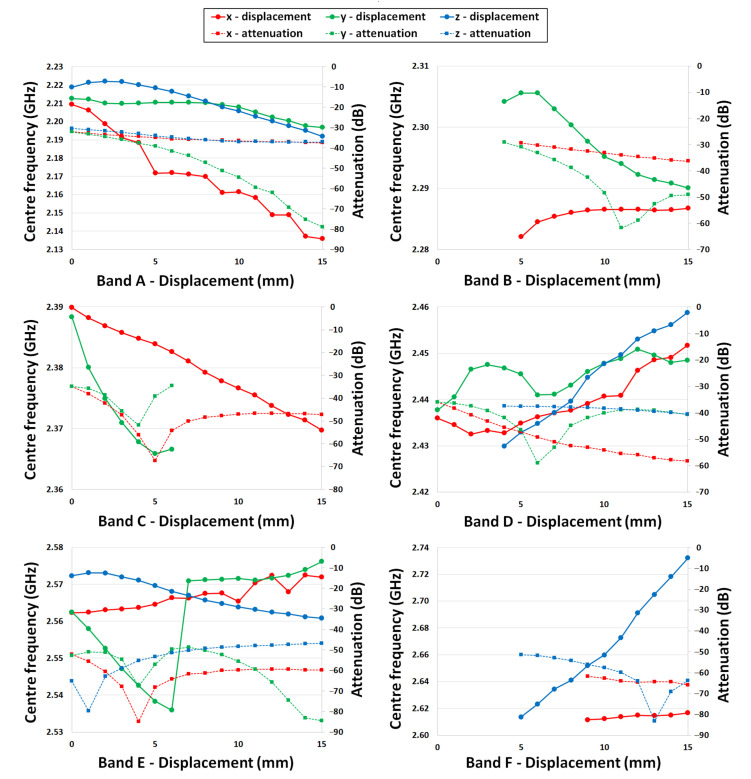
The plots show the frequency (solid line) and attenuation (dotted line) of each feature in bands A to F as x, y and z displacements are introduced between the filters.

When analysing the data, the aim is to find prominent (deeply attenuated) features whose resonant frequencies are sensitive to displacement in ideally only one direction. The results of fitting each of these features for the range of displacements in the x, y and z directions is shown in Figure 5. For cases where the feature was not evident in a particular trace, this data point is missing from the plot.

For band A, the feature was evident but shallow for the majority of x, y and z displacements until the y displacement exceeded 9 mm, from which point the feature quickly deepened. For band B, there was no feature detected for z displacements, and only a shallow feature appearing for large x displacements. However, the feature did become prominent when y displacements exceeded 6 mm. In the frequency range for band C, good displacement-sensitivity was shown at low x and y displacements. whilst being insensitive to the z-axis. At displacements over 6 mm, features were only detected for x displacements. For band D, a weak feature from z displacements could be ignored. There was good sensitivity to x displacements at all but the smallest displacements, but there was potential interference from y displacements in this band in mid-range displacements of 4 mm to 8 mm. Band E was difficult to analyse effectively, as the trace was sensitive to all axes, and with two closely spaced features in this range, mode hopping was evident as displacements varied. Band F provided the best sensitivity for z-axis measurements. It was also insensitive to any y displacements and although there was a reasonably prominent x feature at larger displacements, this occurred at the lower end of this frequency range and would therefore, in practice, be removed through filtering.

**Figure 6:**
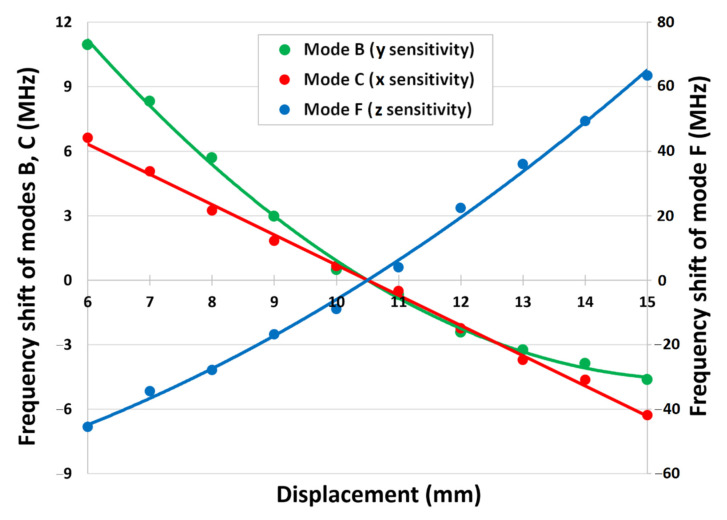
Polynomial fits to the 3 most suitable bands for determining filter displacement indicate x, y, z displacement sensitivities of −1.41 MHz/mm, −1.74 MHz/mm and 12.23 MHz/mm, respectively.

Considering the above analysis, it was decided that band C is suitable for x displacements, band B for y displacements and band F for z displacements, all of which are most effective in the range 6 mm to 15 mm. By resetting the origin to a 10.5 mm offset in the x,y and z directions, this gives the ability of bidirectional-movement sensitivity. Plotting this, as shown in Figure 6, and fitting a second order polynomial (first order in the case of band C) gives displacement sensitivities of −1.41 MHz/mm, −1.74 MHz/mm and 12.23 MHz/mm for x, y and z displacements, respectively. The results are summarized in Table 2.

## 6. Discussion

A comparison of device performance with similar SHM sensors is given in Table 3. The peak sensitivity of this sensor shows a comparable performance to the work of Cho et al. [12] and Yi et al. [13], who reported sensitivities of −26 MHz/mm and −1.19 × 10^4^ ppm/mm (−10.9 MHz/mm), respectively. However, the unique feature of this sensor is the ability to independently measure displacements in all three translational degrees of freedom. In addition, as the sensor is physically composed of two separate pieces, there is no risk of fracturing the device as displacements become significant. Such fracturing is a risk for many RF-based contact sensors.

A point to note in the results is measurement repeatability. In Figure 5, it would be expected that at zero displacement for each trace there are exactly the same conditions, and therefore the same S_21_ trace is expected. However, as can be seen, there is up to a 10 MHz shift at these starting points. Possible reasons for this are a shift in calibration of the VNA as the room temperature changed throughout the day and possible user error in positioning the manual-translation stages. Automating the experimental process to give repeatability in the translational movement of the filters, whilst systematically calibrating the VNA when needed, would be the first step in reducing this variation.

This current work looked at changing x, y and z displacements independently. A future development of this approach will be to assess displacements simultaneously in all directions. A design-of experiments-approach could be adopted, to physically map frequency shifts due to multiple-axis displacements or, alternatively, a full factor analysis at the design and simulation stage undertaken.

The design used here was a simple line-element filter feature rich in notches, originally designed for biosensing applications. There is much scope for design optimization towards specific displacement ranges, and one key change to make this more suited to application would be to design a filter whose features are attenuation peaks as opposed to attenuation valleys, thereby making it easier for a tracking system to monitor frequency changes, particularly for wireless-based arrangements.

## 7. Conclusions

This work has demonstrated the possibility of utilising misaligned line-element filter designs for the detection of translational motion. The main advantage of this approach and contribution to the field of SHM as compared with conventional RF-sensor designs is that it provides independent displacement readings for each translational degree of freedom for the area being monitored. The sensor demonstrated displacement sensitivities up to 12.23 MHz/mm, over a 9 mm range. With suitable arrangement, the sensor may also be applied to strain monitoring. The research community are developing a range of SHM monitoring systems and with the push towards smart cities, such sensor systems will become an integrated part of our lives.

## Figures and Tables

**Table 1:** Designation of features seen in the S_21_ traces.

Band Assignment	Lower Cut-Off (GHz)	Upper Cut-Off (GHz)	Comments
A	2.10	2.25	
B	2.27	2.33	
C	2.33	2.40	
D	2.41	3.50	
E	2.50	2.59	Two bands with mode hopping.
F	2.60	2.80	Visible at higher displacements.

**Table 2:** Summary of results showing sensitivity of features seen in the S_21_ trace due to filter displacement.

Mode	Axis Sensitivity	Working Range (mm)	Frequency at 10.5 mm (GHz)	Sensitivity at 10.5 mm (GHz)	Comments
A	y	9–15			y sensitive > 9 mm, minimal interference from x, z displacements.
B	y	6–15	2.29466	−1.74	y sensitive > 6 mm, insensitive from x, z displacements.
C	x	0–15	2.37603	−1.41	x sensitive over full range, y sensitive at < 6 mm displacements, z insensitive.
D	x	4–15			x sensitive > 4 mm, mid-range y interference, z insensitive.
E	x,y,z	0–15			Position sensitive to movement from all axes.
F	z	6–15	2.66880	12.23	z sensitive > 6 mm. x feature at low end of frequency range.

**Table 3:** Comparison of reported crack- and strain-monitoring sensors.

Sensor Type	Application	Operational Frequency	Sensitivity	Reference
Filter	SHM	2.29 GHz–2.67 GHz	−1.41 MHz/mm (x) −1.74 MHz/mm (y) 12.23 MHz/mm (z)	This work.
Antenna	SHM	2.9 GHz	−26 MHz/mm (crack width) −5.232 kHz/με	Cho et al. [12]
Antenna	SHM	913 MHz	−1.19 × 10^4^ ppm/mm (crack length) −0.893 ppm/µε	Yi et al. [13]
Antenna	SHM	17.2 GHz, 20.5 GHz	16.4 kHz/µε (width) 17.2 kHz/µε (length)	Tata et al. [22]
Antenna	Wearables	3 GHz	40 dB/ε	Rakibet et al. [21]
Antenna	Wearables	1.6 GHz	51.4 %/ε	Kim et al. [16]
Antenna	SHM	1.5 GHz	54.6 %/ε	Daliri et al. [24]
Antenna	Wearables	955 MHz	16.7 dB/ε	Merilampi et al. [20]
Antenna	SHM	920 MHz	0.69 dB/mm	Cazeca et al. [18]
Antenna	SHM	918 MHz	−852 Hz/με	Dan et al. [2]
Antenna	Wearables	915 MHz	46.3 %/ε	Hasani et al. [19]
SAW	SHM	915 MHz	−1.80 ppm/µε	Humphries et al. [11]
Antenna	SHM	909 MHz	0.55 kHz/µε	Kuhn et al. [1]
Antenna	SHM	867 MHz	150 deg/mm	Caizzone et al. [17]
SAW	SHM, Environmental	40 MHz	0.07 deg/µε	Saitoh et al. [10]
Strain gauge	SHM	24.4 KHz	2 Hz/µε	DiGiampaolo et al. [3]

## Data Availability

The data presented in this study are openly available in data.ncl at doi: 10.25405/data.ncl.21362793 (accessed on 15 November 2022).
